# Electrical properties of a liquid crystal dispersed in an electrospun cellulose acetate network

**DOI:** 10.3762/bjnano.9.18

**Published:** 2018-01-15

**Authors:** Doina Manaila Maximean, Octavian Danila, Pedro L Almeida, Constantin Paul Ganea

**Affiliations:** 1 University Politehnica of Bucharest, Department of Physics, 313 Spl. Independentei, 060042, Bucharest, Romania; 2 I3N - CENIMAT, Departamento de Ciência dos Materiais, Faculdade de Ciências e Tecnologia, FCT/UNL, 2829-516 Caparica, Portugal; 3 Área Departamental de Física, ISEL, Instituto Superior de Engenharia de Lisboa, I.P.L., R. Conselheiro Emídio Navarro, 1959-007 Lisboa, Portugal; 4 National Institute of Materials Physics, POBox MG 07, 077125 Magurele, Romania

**Keywords:** cellulose nanocomposite, dielectric spectroscopy, impedance spectroscopy, liquid crystal, optical transmission

## Abstract

Electro-optical devices that work in a similar fashion as PDLCs (polymer-dispersed liquid crystals), produced from cellulose acetate (CA) electrospun fibers deposited onto indium tin oxide coated glass and a nematic liquid crystal (E7), were studied. CA and the CA/liquid crystal composite were characterized by multiple investigation techniques, such as polarized optical microscopy, dielectric spectroscopy and impedance measurements. Dielectric constant and electric energy loss were studied as a function of frequency and temperature. The activation energy was evaluated and the relaxation time was obtained by fitting the spectra of the dielectric loss with the Havriliak–Negami functions. To determine the electrical characteristics of the studied samples, impedance measurements results were treated using the Cole–Cole diagram and the three-element equivalent model.

## Introduction

The widely known polymer dispersed-liquid crystals (PDLCs) are a class of liquid crystal (LC)-based electro-optical devices, formed by LC droplets dispersed in a solid polymeric matrix [1–5]. The optical transmission of such devices is sensitive to external AC electric fields. Without external influence (the so called OFF state), PDLCs scatter the incident light due to the fact that the LC molecules are anchored to the inner surface of the droplets in the polymeric matrix, having a non-uniform orientation between different droplets. Since there is no uniform direction of alignment of the LC director between different droplets, the optical path of the incident light travelling through a PDLC is different from point to point, due to the mismatch between the effective refractive index of the LC and the refractive index of the polymer. The state where the device becomes transparent (the so called ON state) can be achieved by applying an electric field with adequate magnitude. Under the action of the field, the LC molecules inside each droplet align along the direction of the field, and the ordinary refractive index of the LC becomes equal to the refractive index of the polymeric matrix, creating a constant optical path for the incident light along the surface of the PDLC sample, reducing the light scattering to a minimum and increasing the transmission of light to a maximum. PDLC films have many interesting applications in optoelectronics (light valves, polarizers), architectural windows, reduction of solar heat load [2,6], nonlinear optics [7–9] and nanotechnological applications [10–20].

**Figure 1:**
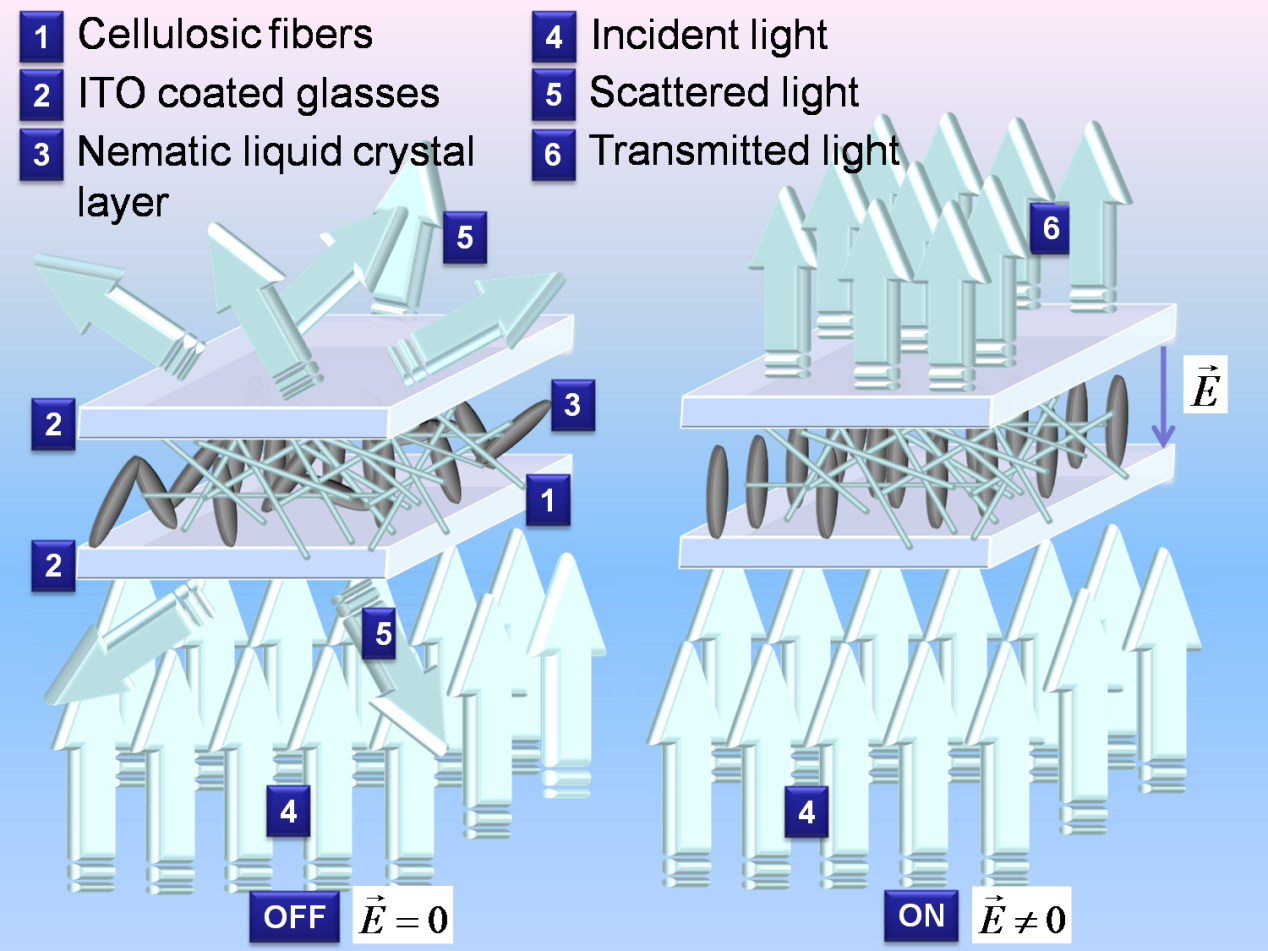
Schematic representation of morphology and working principle of the electrospun cellulose network with the dispersed liquid crystal. The applied electric field is denoted by 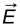.

The macroscopic electro-optical effect in electrospun cellulose acetate/LC composites is similar to the one observed in traditional PDLC. The major difference is that instead of having small droplets of LC confined in a polymeric matrix, the LC fills the voids between fibers in mats of non-woven electro-spun cellulose acetate fibers [21–27]. The electrospun cellulose fibers were deposited on indium tin oxide (ITO)-coated glass, and two such fiber-coated glass plates form a sandwich type cell, where the nematic liquid crystal (NLC) is filled in by capillarity [21–22,28]. A schematic representation of the electrospun cellulose network with the dispersed liquid crystal as well as the working principle is given in Figure 1.

The cellulose acetate used in the preparation of the CA/LC samples [29–33] was obtained from green and renewable sources. The used nematic LC is E7, a mixture of alkylcyanobiphenyls with a cyano head group [34–36]. This paper presents the investigations of the electrical properties of an electrospun CA network with dispersed liquid crystals. Dielectric spectroscopy (DS) was performed over wide ranges of frequency and temperature to determine physical properties such as activation energy and characteristic time. The experimental results of DS were fitted to the Havriliak–Negami [37] model and further modelled by the Vogel–Fulcher–Tammann law. In addition to this, impedance spectroscopy measurements were performed, and the results were processed using a simple equivalent circuit model, that permits the extraction of electric circuit parameters.

## Results and Discussion

### Scanning electron microscopy

**Figure 2:**
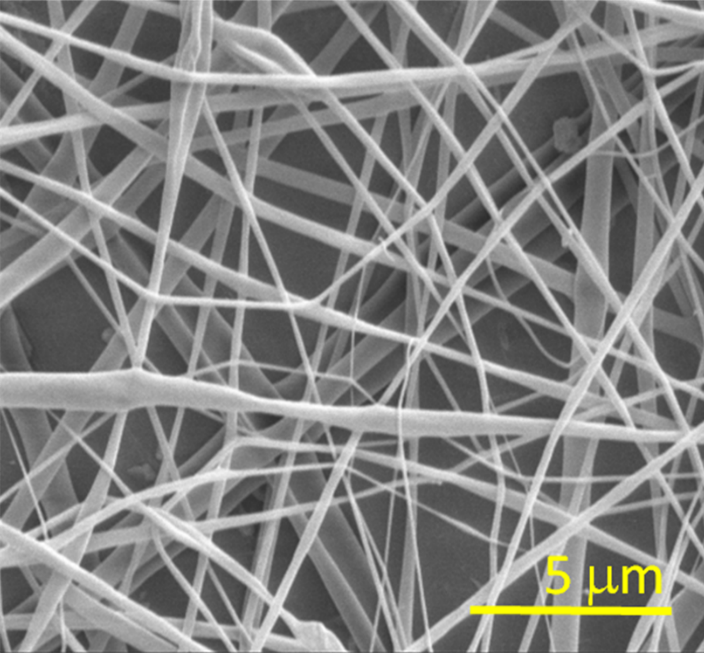
SEM image of deposited electrospun CA fibers.

Figure 2 shows the SEM image of the deposited fibers [21–22,28]. In the SEM image, the fibers exhibit a wide dimension range, starting with dozens of nanometres.

### Polarized optical microscopy

**Figure 3:**
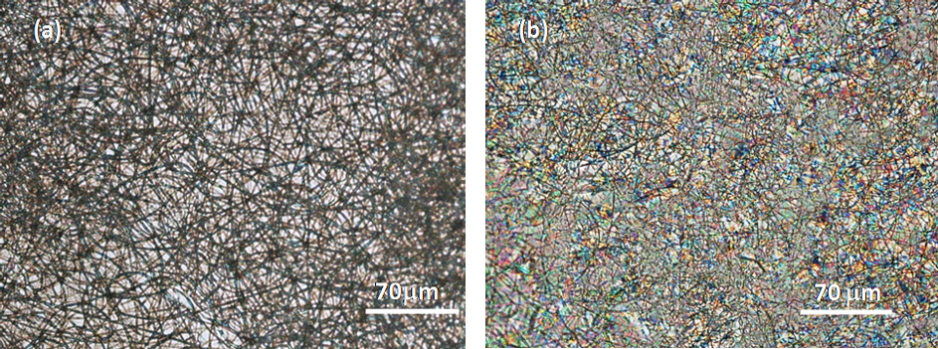
Polarizing optical microscopy images of the electrospun CA a) without LC and (b) filled with E7.

Figure 3 presents the polarizing optical microscopy images of the electrospun CA cell, (a) without LC and (b) filled with E7, taken between crossed polarizers.

As it can be perceived by the colors seen in the POM image, the LC fills the voids between the CA fibers (Figure 3b). The multiplicity of colors arises from the existence of liquid crystal polydomains imposed by the distortion of the nematic director field through the fibers.

### Dielectric spectroscopy

The characterized samples were electrospun CA without LC, and the same CA sample after filling in the LC (denoted as CA/E7 composite). The DS measurements were carried out under isothermal conditions, in the frequency range from 10^−1^ to 10^7^ Hz, in a temperature domain from 293 to 350 K.

The DS results were obtained by plotting the real and imaginary components of the complex permittivity function ε*(ω) = ε′(ω) − iε″(ω). Here, ε*(ω) is the dielectric permittivity, the real part, ε′(ω), is the dielectric constant, and the imaginary part, iε″(ω), is the dielectric loss [37].

**Figure 4:**
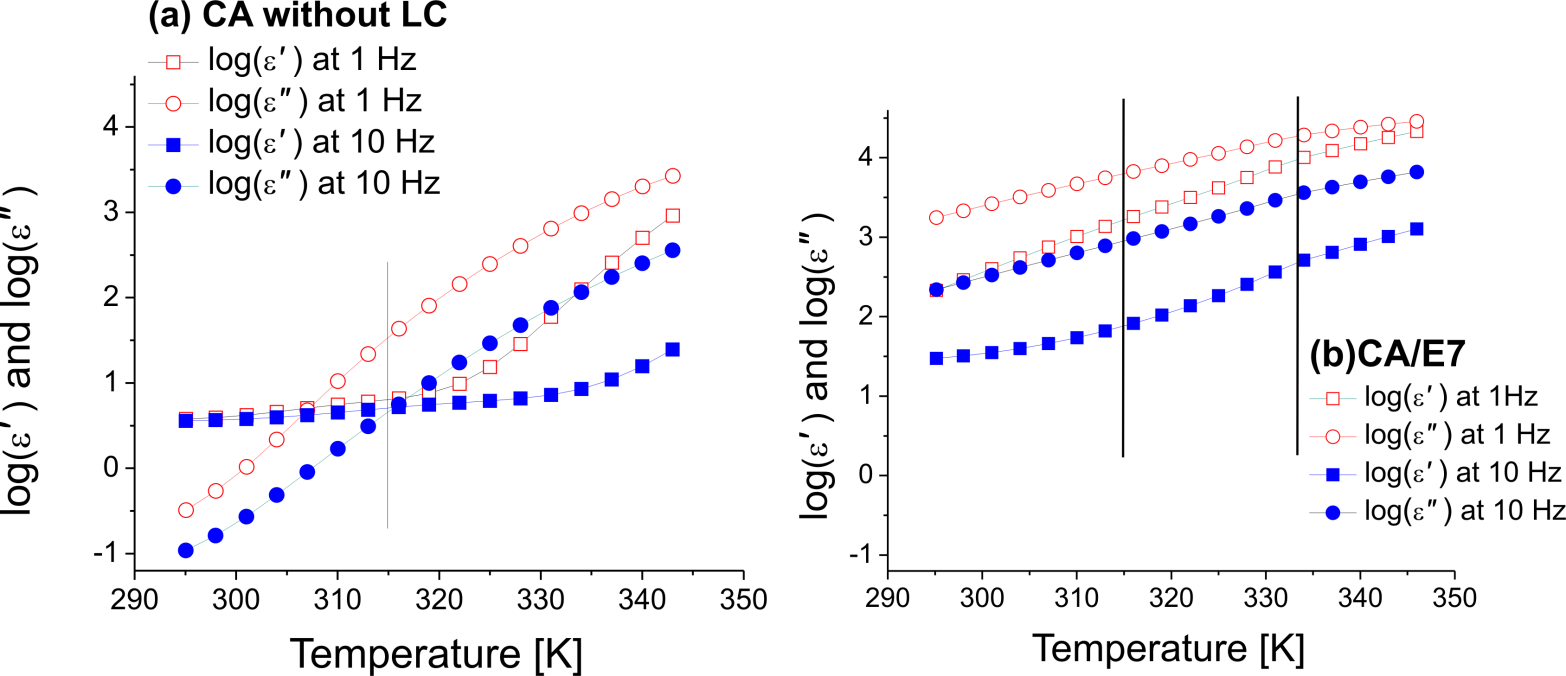
Dielectric constant and dielectric loss (logarithmic scale) as functions of the temperature for (a) the CA cell before filling in the LC and for (b) the CA/E7 cell, at 1 Hz and 10 Hz.

Figure 4 shows dielectric constant and dielectric loss as functions of the temperature at two constant representative frequencies, 1 Hz and 10 Hz, for (a) the cell with CA fibers before filling in the LC, and (b) for the same cell after the LC was introduced. The changes of slope may indicate phase transitions. Thus, we can suppose that CA without LC might have a phase transition at *T* = 315 K. Similarly, the sample CA/E7 might have a phase transition at about 333 K. The verification of this supposition is based on the fitting parameters of the Vogel–Fulcher–Tammann law (see below in Figure 6 and Table 1).

**Figure 5:**
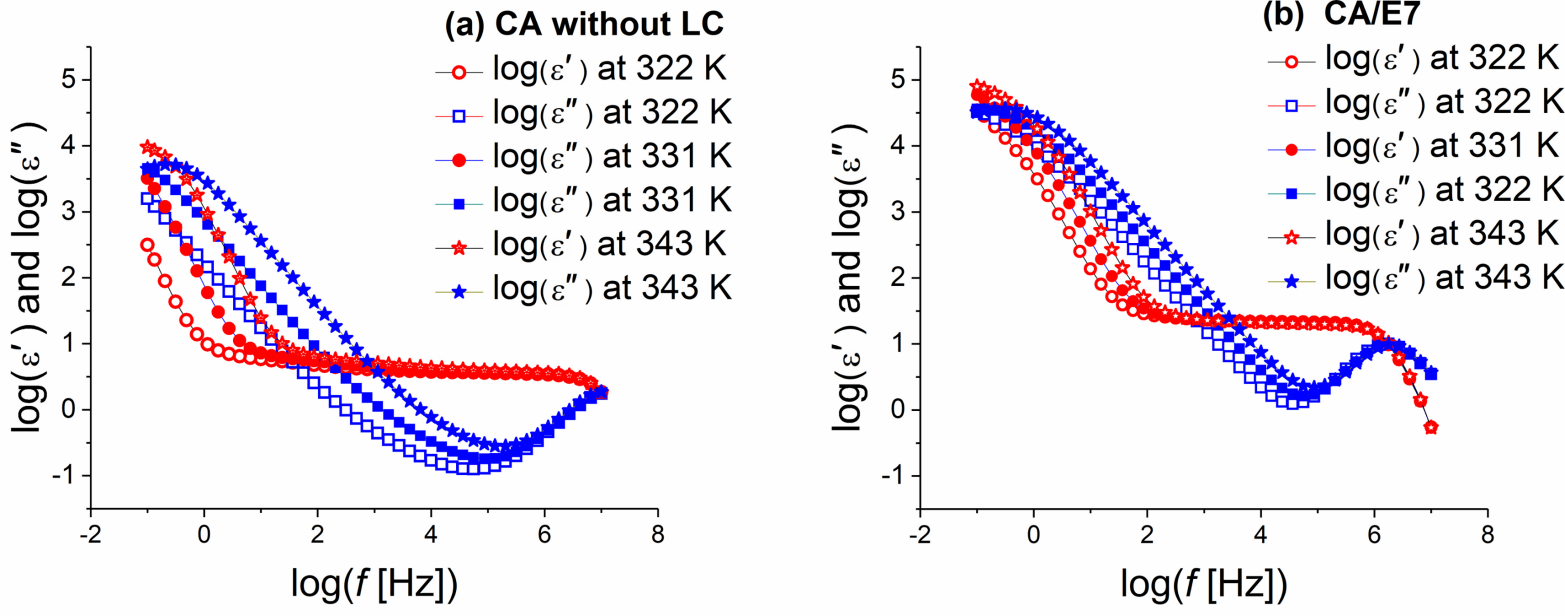
Dielectric constant and dielectric loss (logarithmic scale) as functions of the frequency for (a) the CA cell before filling the LC and for (b) the CA/E7, at three different temperatures.

Figure 5 presents dielectric constant and dielectric loss as functions of the frequency for the CA cell (a) before and (b) after filling in the LC, at three constant temperatures. For the CA sample without LC measured in the high frequency (HF) domain, one notices a relaxation process outside the measurement domain, while in the low frequency (LF) range, two CA-attributable almost overlapping relaxation processes can be observed at 1 Hz and 1000 Hz. The CA/E7 sample exhibits a relaxation process due to the LC in the HF measurement range, and two almost overlapping relaxation processes in the LF range.

**Figure 6:**
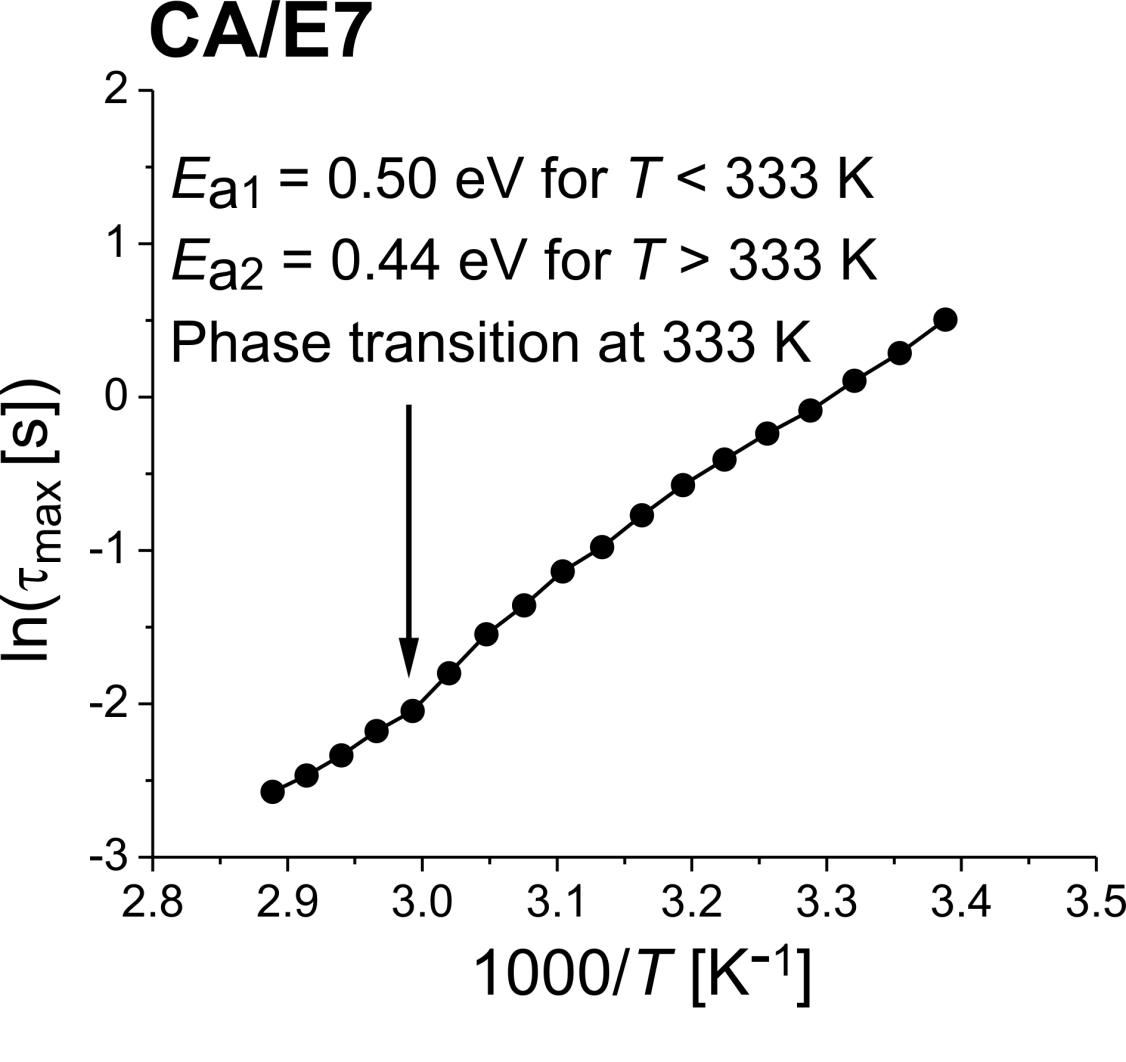
Relaxation time as a function of the inverse of temperature for the CA/E7 sample.

The characteristic relaxation times were obtained by fitting the spectra of the dielectric constant and dielectric loss with the Havriliak–Negami function [37–40]:

[1]
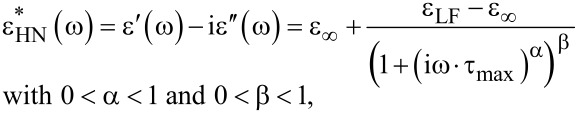


where ε′(ω) is the dielectric constant and ε″(ω) is the dielectric loss, ε_LF_ is the low frequency permittivity, ε_∞_ is the permittivity in the HF limit, and τ_max_ is the characteristic relaxation time of the medium. Figure 6 presents the characteristic relaxation time as a function of the inverse of temperature for the cellulose acetate sample with LC.

In the temperature range between 293 K and 350 K, where the DS measurements were performed, the pure liquid crystal E7 exhibits an anomalous behavior of the nematic phase around 305 K [26]. This behavior cannot be seen from the results presented here, since the CA component of the composite system has an important influence on the values of dielectric permittivity (Figure 5). Moreover, because of the interaction with the surface of the CA fibers, the dynamics of the E7 molecules is more or less attenuated, as compared to the pure LC. Thus, as in other composite systems, the phase transitions can be modified or even suppressed [38–40].

The dependency τ_max_ = *f*(1/*T*) can be modeled using the Vogel–Fulcher–Tammann (VFT) law:

[2]
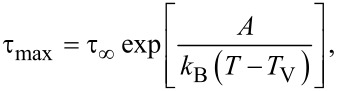


where *A* is a material constant, *k*_B_ is Boltzmann’s constant, *T* is the measured temperature, *T*_V_ is the Vogel temperature, and τ_∞_ is a pre-exponential factor. The results are summarized in Table 1.

**Table 1:** Extracted values of the DS characteristic constants for the CA/E7 .

no.	sample type	investigation method	temperature range [K]	activation energy, *E*_a_^a^ [eV]	Vogel temperature *T*_V_ [K]	relaxation time, τ_max_ [s]

1	CA/E7	DS, Vogel–Fulcher–Tammann	295–328	0.50	0.61	3.79·10^−9^
			333–346	0.44	0.22	2.38·10^−8^
2	CA/E7 [23]	*I*–*V* curves, Arrhenius	308–330	0.66	—	—
			333–353	0.54	—	—

^a^Because the obtained Vogel temperature is very small, the VFT equation approximates an Arrhenius-like expression, the material constant *A* being incorporated into the activation energy *E*_a_.

The VFT data processed parameters are presented parallel to the previously published results obtained using the *I*–*V* curves and the Arrhenius formula [28]. A good agreement of the obtained activation energies is observed, with lower values in the high-temperature domain, after the nematic-to-isotropic phase transition.

### Impedance spectroscopy

The impedance, *Z*, and the quality factor (dielectric loss tangent), θ, were measured using impedance spectroscopy [41–43]. Based on the obtained data, the real (active) impedance component, Z′, and the imaginary (reactive) impedance component, Z″ impedance components were calculated using the equations

[3]
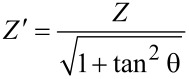


and

[4]
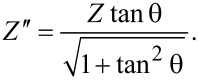


**Figure 7:**
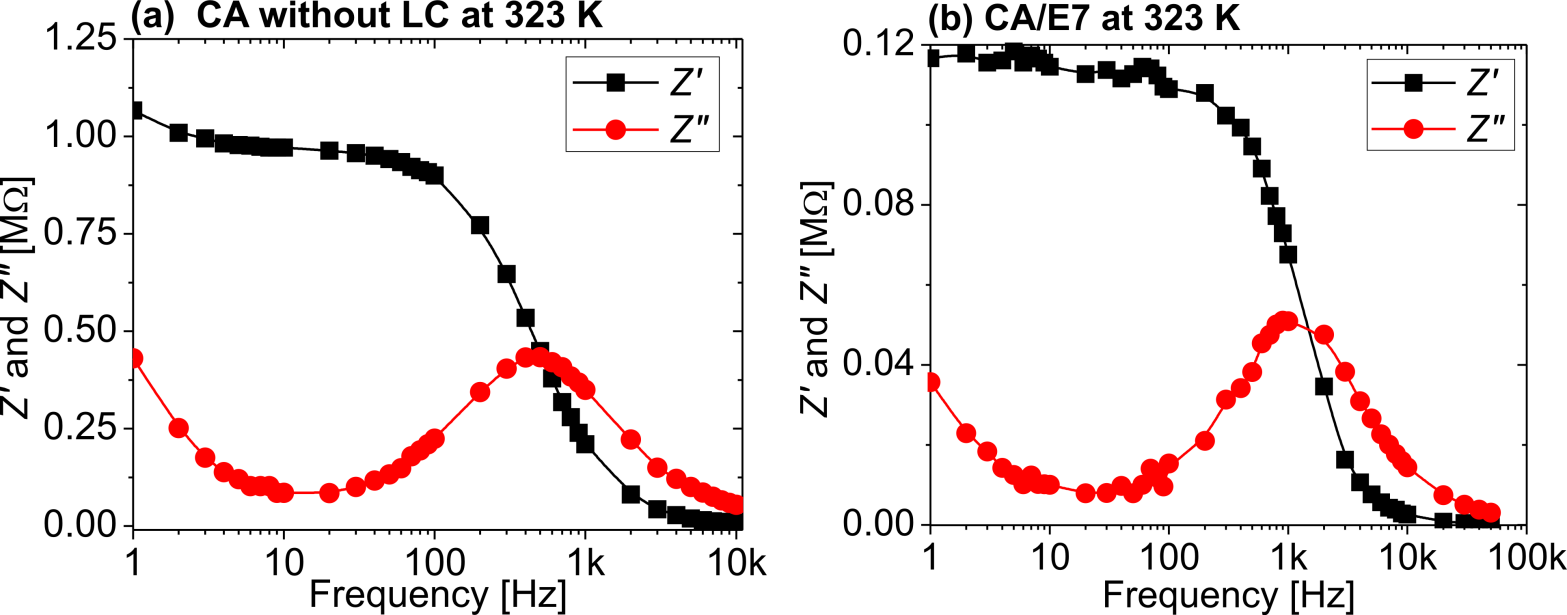
Active and reactive part of the impedance as functions of the frequency, for (a) the CA cell and for (b) the CA/E7 sample, at 323 K.

**Figure 8:**
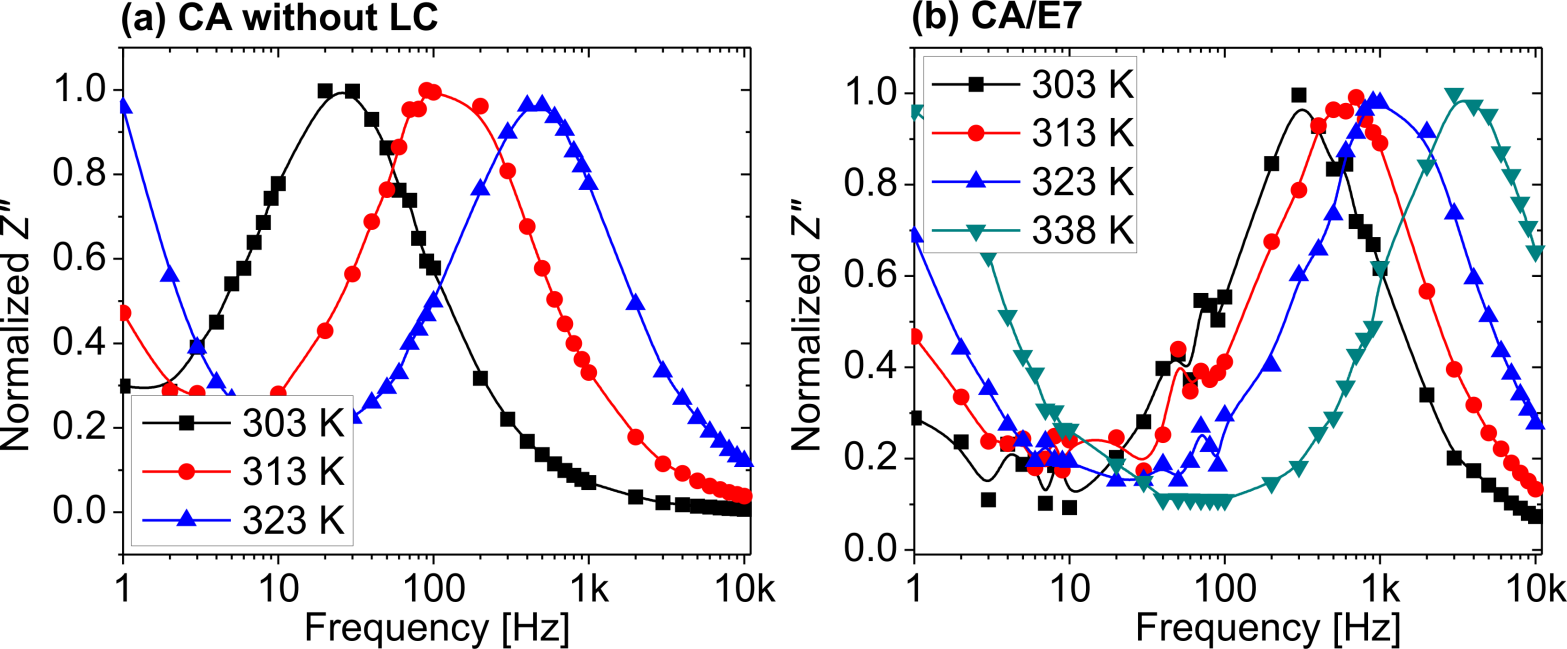
Normalized reactive part of the impedance as a function of the frequency for (a) the CA cell and for (b) the CA/E7 sample, at different temperatures.

Figure 7 presents selected plots of the active and reactive part of the impedance as functions if the frequency, for (a) the CA cell and for (b) the CA/E7 samples, at 323 K. The curves were obtained by standard interpolation. The reactive part of the impedance plays an important role in determining the components of the equivalent electrical model, and it is presented in Figure 8 for different temperatures. The curves were obtained by interpolation of the raw data.

The spectra of the two impedance components, *Z*′ and *Z*″, are different for the samples CA and CA/E7. Similar to the DS results, at low frequencies an E7 molecule-dynamic dipolar relaxation process is overlapping on the intrinsic CA processes (Figure 7b and Figure 5b). Another process is observed at high frequencies (1 MHz), attributed to LC molecules in the bulk, “far” from the CA fibers, as seen in Figure 5b. The slow process noticed at low frequencies (100 Hz), which is observed in Figure 7b and less evident in Figure 5b, is due to the additional interaction of dipoles with the surface. As the temperature increases, the peak values of the reactive impedance shift to higher frequencies (Figure 8).

**Figure 9:**
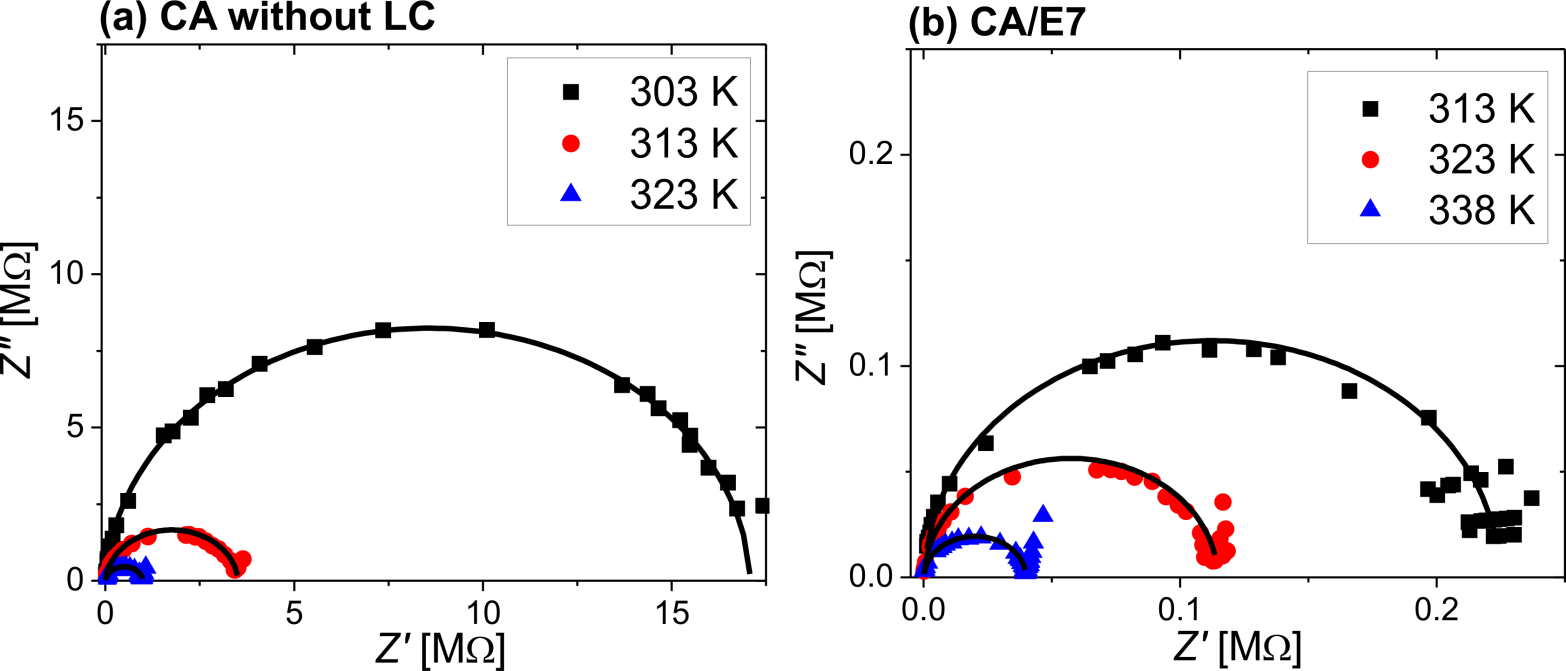
Cole–Cole diagrams for (a) the CA cell at 303 K (black solid squares), 313 K (red solid circles), and 323 K (blue solid upward triangles), and for (b) the composite CA/E7 at 313 K (black solid squares), 323 K (red solid circles), and 338 K (blue solid upward triangles).

**Figure 10:**
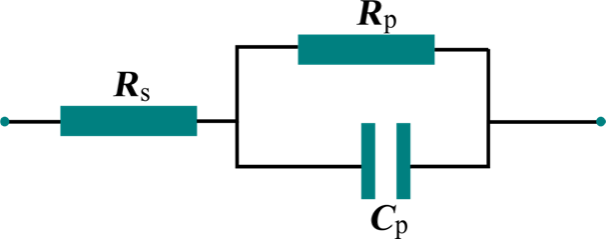
Equivalent three-element model circuit, formed by a serial resistance, *R*_s_, a parallel resistance, *R*_p_, and a parallel capacitance, *C*_p_.

The Cole–Cole [32] diagrams, *Z*″ = *f*(*Z*′), are presented in Figure 9. The semicircular shapes of the diagrams allow for modelling the raw data with a theoretical three-element electric circuit model, consisting of a series resistance, a parallel resistance and a parallel capacitor. The model is presented in Figure 10.

The values of the serial and parallel resistances can be calculated directly from the Cole–Cole diagrams using the equation:

[5]
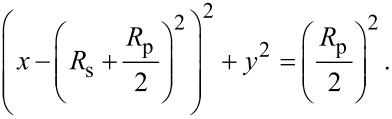


To determine the parallel capacitance for the two samples, we extracted relevant information from the frequency plot of *Z*″ (Figure 8) and the Cole–Cole diagram (Figure 9). In the Cole–Cole diagram, for this particular model, the coordinate having a maximum *Z*″ is also the position at which ωτ = 1, where ω is the angular frequency. This angular frequency can be deduced by estimating the frequency at which the maximum value of *Z*″ is obtained (Figure 8). Also, the electrical response time is τ = 1/(*R*_p_*C*_p_), and knowing the angular frequency, the capacitance can be determined. Based on the fitting technique, we modeled the sample behavior by a simple three-element electric circuit (Figure 10), which describes a single relaxation process. The values of the determined electric parameters have an appropriate temperature variation (Table 2).

**Table 2:** Characteristic elements of the equivalent three-element circuit, extracted from the Cole–Cole diagrams.

no.	*T* [K]	sample	*R*_S_ [kΩ]	*R*_p_ [MΩ]	τ [s]	*C*_p_ [nF]

1	313	CA	1810	714	0.5495	0.769
2	313	CA/E7	15.17	7.37	0.3616	49
3	323	CA	2350	1350	0.2936	0.218
4	323	CA/E7	17.58	12.8	0.3331	26
5	338	CA/E7	29.37	18.2	0.1667	9.1

### Electro-optical measurements

**Figure 11:**
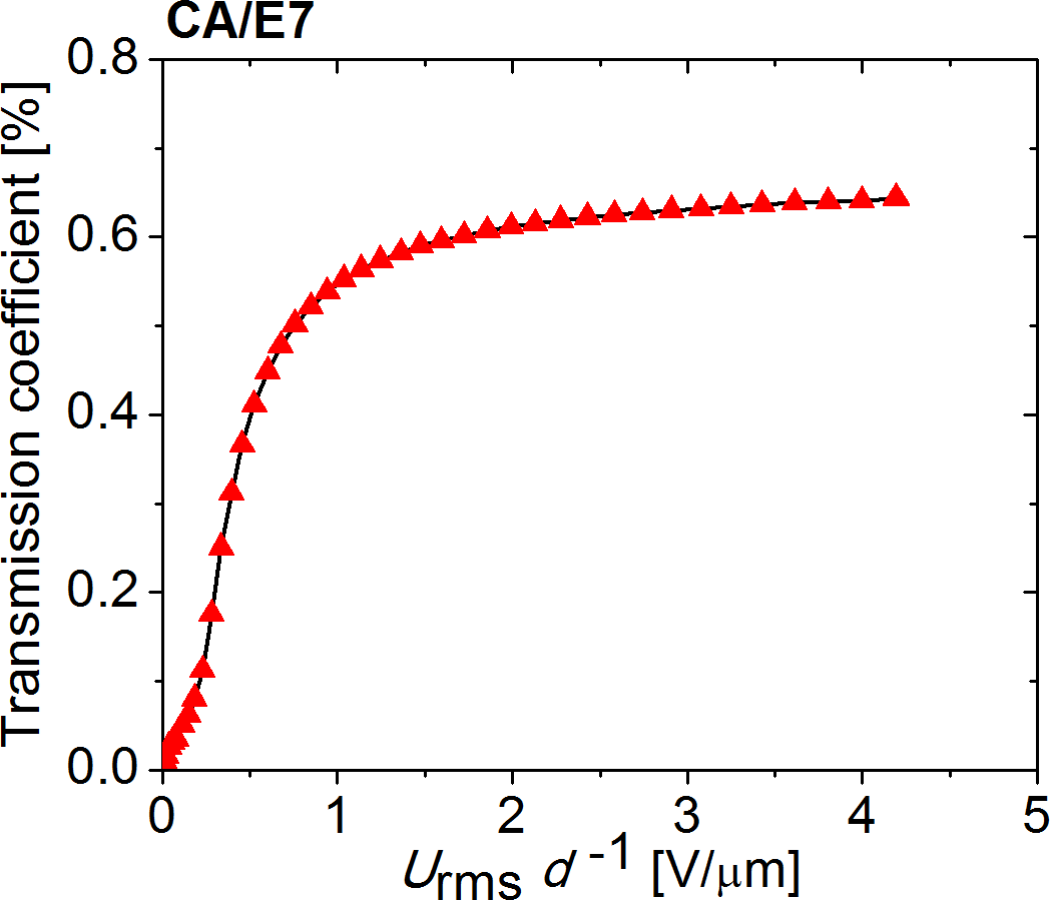
Optical transmission of the sample CA/E7 as a function of the applied ac electric field.

The optical transmission was measured using the setup described in the Experimental section of this paper and in [21–22,28]. The transmission coefficient is defined as the ratio between the light intensity passing through the sample and the incident light intensity. Figure 11 presents the transmission coefficient versus the ac electric field. An improved characteristic is observed, as compared to the previous similar devices [21–22,28], with a stable “ON” state and a lower required electric field to switch between “OFF” and “ON”, at values of 1–1.5 V/μm. No significant optical hysteresis was observed between the transmission curves obtained when increasing and decreasing the applied voltage. The electro-optical response remained stable when repeating the switching cycles.

## Conclusion

CA electrospun nanofibers were deposited onto ITO-coated glass and an electro-optic cell was formed by two such glass plates with fibers in between. By filling in the nematic liquid crystal E7 a light scattering device with a polymer-dispersed liquid crystal was obtained. Dielectric spectroscopy (DS) and impedance measurements were performed on the electro-optic cells before and after filling in the LC. Also, the dependency of the dielectric constant and electric energy loss on frequency and temperature was studied. The nematic–isotropic phase transition temperature of E7 and the activation energy were determined, and found to be in good agreement with previously obtained data. The relaxation time was obtained by fitting the spectra of the dielectric loss with the Havriliak–Negami function. Impedance measurements were evaluated using Cole–Cole diagrams and the three-element equivalent model, which permits the estimation of the equivalent resistances and capacity, necessary in practical applications. To test the efficiency of the electro-optic device, optical transmission measurements in external ac fields were performed.

## Experimental

### Sample preparation

**Figure 12:**
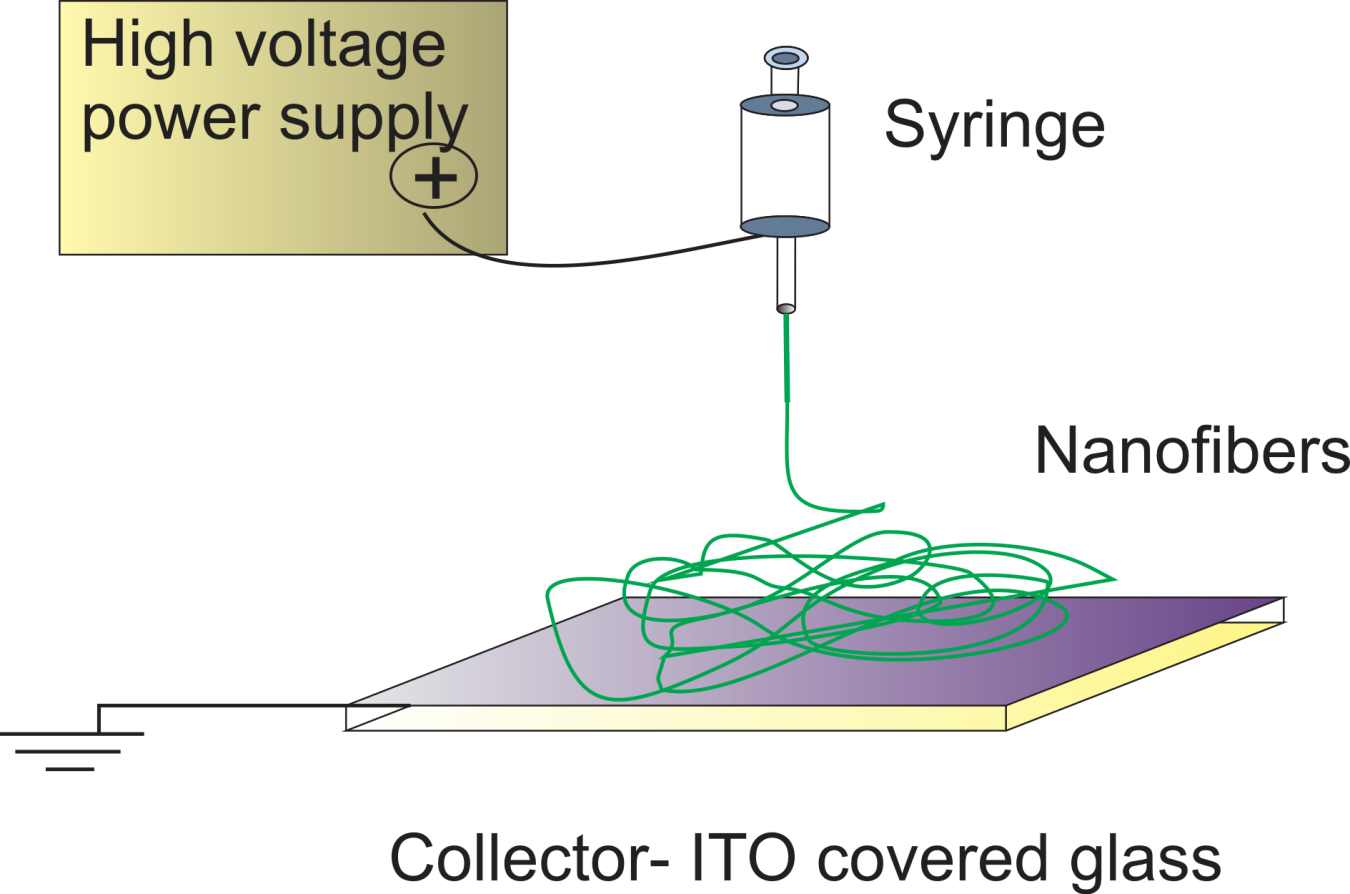
Schematic representation of electrospinning cellulose fibers from solution.

The non-woven nano- and micro-fiber cellulose mats used to prepare the electro-optical cells were produced by electrospinning [21–22,28] from an isotropic solution (15%) of cellulose acetate (CA, Aldrich, *M*_W_ = 60.000 g·mol^−1^_,_ 40% acetyl groups) in a mixture of dimethylacetamide/acetone (1:2). The solution was prepared at room temperature. After the first week, it was stirred every day and kept away from light for at least four weeks until used. To produce the fibers, the solution was poured into a 1 mL syringe (diameter 4.5 mm) fitted with a 27-gauge needle (diameter 0.2 mm), which was then placed on the infusion syringe, pump (KDS100) to better control the incoming flow of the polymer solution. A conducting ring is held coaxially with the needle tip. The needle and the ring were directly connected to the positive output of a high-power voltage supply (Glassman EL 30 kV), as schematically presented in Figure 12. After applying the electric potential between the metallic syringe tip and the plate, the fibers were deposited directly onto the ITO-coated glass, over the ITO surface. The fibers were then carefully dried in vacuum, at room temperature, for 72 h before further characterization and use.

To prepare a sample cell, two ITO-coated glasses with fibers were glued together with the fiber mat acting as a spacer. After assembly, the cells thickness between the two ITO-coated glasses was of around 20 μm. This sample was named CA. Before filling the cells with the liquid crystal, the CA cells were kept at 110 °C for 30 min and then allowed to cool slowly to 100 °C. The nematic liquid crystal E7 (commercially available from Merck) was then filled in through capillarity. The liquid crystal E7 is a mixture of alkylcyanobiphenyls with a cyano head group, exhibiting a nematic to isotropic transition at 333.5 K. The refractive index of the CA is 1.45 and the ordinary refractive index of E7 liquid crystal is 1.51. This sample type was named CA/E7.

### Characterization techniques

#### Scanning electron microscopy

The fiber dimensions and distribution were characterized by SEM, using a SEM DSM 962 model from Zeiss Company after thermal evaporation under vacuum of gold onto the surface of the fibers.

#### Dielectric spectroscopy

The dielectric spectroscopy measurements were performed using a broadband dielectric spectrometer, NOVOCONTROL, consisting of two devices: an Alpha-A high-performance frequency analyzer in the LF domain (0.01 to 10^7^ Hz) and an Agilent E4991A RF impedance/material analyzer for the HF range, (1 MHz to 3 GHz), equipped with WinDETA software. The temperature was controlled within 0.2 K, at a constant ac voltage of 0.5 V.

#### Impedance measurements

Impedance spectroscopy was performed using a high-resolution LCR meter, Hioki – 3200-50 in the frequency range from 1 Hz to 10 kHz and a temperature-controlled hot-stage Mettler-Toledo 3200 series.

#### Electro-optical transmission

**Figure 13:**
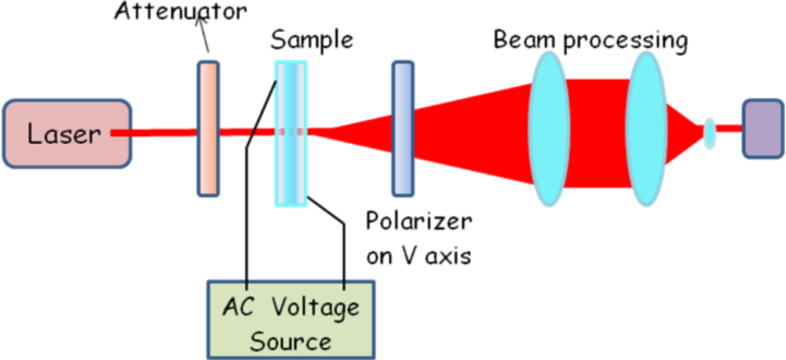
Experimental set-up for the optical transmission measurement.

The optical transmission was measured using the setup previously described [21–22,28] and presented in Figure 13. A He–Ne laser beam (wavelength 623.8 nm) passes through the sample, which is modulated by an ac voltage provided by a function generator–amplifier system. The laser beam is detected by a high-speed photodiode with adjustable gain (Thorlabs). The electrical signal generated by the photodiode was recorded with a high-resolution voltmeter Keithley 6517A.
